# Innovative Strategies: Use of Stromal Cell-Derived Secretome for Chronic Wound Therapy

**DOI:** 10.3390/ijms26125609

**Published:** 2025-06-11

**Authors:** Daniela-Madalina Ghetu, Karine Raymond, Irina Titorencu, Maya Simionescu

**Affiliations:** 1 Institute of Cellular Biology and Pathology “Nicolae Simionescu”, 050568 Bucharest, Romania; madalina.ghetu@icbp.ro (D.-M.G.); maya.simionescu@icbp.ro (M.S.); 2 Department of Anatomy and Embryology, Leiden University Medical Center, 2300 RC Leiden, The Netherlands; k.i.raymond@lumc.nl; 3 The Novo Nordisk Foundation Center for Stem Cell Medicine (reNEW), Leiden University Medical Center, 2300 RC Leiden, The Netherlands; 4 University of Grenoble Alpes, CEA, Inserm, IRIG, UA13 BGE, Biomics, 38000 Grenoble, France

**Keywords:** chronic wounds, secretome, mesenchymal stromal cells, fibroblasts, wound models

## Abstract

Chronic wounds represent a major therapeutic challenge, with limited effective treatment options currently available. Both cellular and acellular approaches are being explored to address this issue, with mesenchymal stromal cells (MSCs) emerging as a promising option. While these cells have been extensively studied, alternative stromal cell sources, such as fibroblasts (Fbs), may also possess comparable therapeutic potential. Thus, this review focuses on stromal cell-derived secretomes (conditioned medium) as a source of acellular therapy for chronic wounds and presents the available wound-healing models (in vitro, ex vivo, and in vivo) suitable for evaluating their therapeutic efficacy, prior to clinical application. By conducting an analysis of the existing studies, we present the impact of the cell culture conditions on the enhancement in the bioactivity of the MSC/Fb-derived conditioned medium, a research area that continues to evolve.

## 1. Introduction

Generally, chronic wounds are characterized by their persistence for more than three months and are often associated with comorbidities such as diabetes mellitus and vascular insufficiency; they imply, among other things, impaired blood circulation [1]. Despite extensive efforts directed towards the prevention and treatment of chronic wounds, they pose a significant global burden [2] with a substantial economic, psychological, and physical impact [3].

Diabetes, a growing health concern, is the leading cause of non-healing ulcers, with its prevalence increasing in recent years [4]. Diabetic wounds, which are particularly challenging to treat, can lead to severe complications such as limb amputations that severely impact the quality of life for a significant number of the global population [5]. Moreover, it is estimated that diabetic wounds account for 25–50% of the total cost associated with diabetes treatment [6].

The complex pathophysiology of chronic wounds has made drug development particularly challenging. To date, no specific pharmacological treatment has been proven effective in wound healing. Various treatment approaches, such as different wound dressings, are available to improve the healing process. However, they are often associated with high costs and frequently result in unfavourable outcomes. Consequently, overcoming the current barriers in wound care by developing more effective and cost-efficient therapeutic strategies is a significant focus of ongoing research.

Recent findings consist of the reporting of a hydrogel with immunoregulatory properties that scavenges ROS, promotes macrophage M1-to-M2 transition, reduces inflammation, and enhances oxygen availability via near-infrared-induced hyperthermia in hyperglycaemic wound environments [7]. As the construct facilitated decreased inflammation, enhanced collagen synthesis, and faster blood vessel regeneration, the study introduced a safe innovative method for the effective management of diabetic chronic wounds.

Over the past two decades, cell therapies, particularly mesenchymal stromal cell (MSC)-based therapies, have emerged as a promising option for the treatment of chronic wounds. MSCs are considered attractive therapeutic agents due to their ease of isolation, high differentiation capacity, and lack of significant immunogenicity and ethical concerns [8]. Moreover, regenerative medicine approaches offer significant advantages over conventional treatment, which primarily focuses on wound closure rather than addressing the underlying biological mechanisms.

More recently, acellular therapy based on the secretome collected from cultured MSCs has been developed as a promising option for wound healing, showing a greater therapeutic effect over the use of the cells themselves. This effect is attributed to the paracrine activity of bioactive molecules such as growth factors (GFs) and cytokines, which are either directly secreted or carried by extracellular vesicles (EVs) such as exosomes [9]. Moreover, acellular therapy based on the MSC-derived secretome offers several additional advantages: it mitigates the risks associated with cell transplantation, such as immunogenicity and tumorigenicity [10], and has ease of storage and a longer shelf life [11], features important for clinical applications.

Recently, multiple studies have shown that many similarities exist between MSCs and fibroblasts (Fbs), the cells abundantly found in the connective tissues of most organs [12,13,14]. Thus, Fbs can serve as an alternative to MSCs, as they are easier to isolate in significant quantities from various tissues such as the skin (dermis) and can be more readily maintained in vitro [15].

In this review, we focus on the available data regarding the therapy of chronic wounds based on MSC- and Fb-derived secretomes. In addition, an overview of in vitro and in vivo models suitable for testing these approaches is provided.

## 2. MSCs and MSC-Derived Secretome Can Be Employed for Wound-Healing Therapy

### 2.1. Overview of MSCs

First isolated and described by Friedenstein et al. [16], MSCs have become widely studied as promising candidates for cell-based therapies for regenerative medicine. Their main feature is the ability to self-renew and differentiate into mesodermal lineage cells, such as osteoblasts, chondrocytes, and adipocytes. This trilineage differentiation potential is often used in vitro to confirm the identity of MSCs [17]. However, some reports have indicated that MSCs can also differentiate into non-mesenchymal lineages, such as epidermal and neuronal cells [8]. Other attributes, such as the ease of harvest and low immunogenicity, make these cells a safe and effective candidate for various therapies [18]. Bone marrow (BM) and adipose tissue are the most frequently used adult donor tissues for MSCs [19].

Due to the pressing medical need to identify new therapeutics that can effectively accelerate skin wound closure, MSC-based therapy has emerged as an attractive option [20]. Several clinical trials have been conducted to assess the safety and efficacy of stromal cell-based therapy in wound healing and skin regeneration, with BM-derived MSCs (BM-MSCs) and adipose-derived stromal cells (ADSCs) being the most commonly used MSCs [21,22]. Regarding chronic wounds, most of the evidence for the role of MSCs in the healing process comes from using animal models, particularly rodents; only a limited number of clinical studies have been reported.

### 2.2. Constituents of MSC-Derived Secretome

It was shown that stromal cells transplanted to the site of injury act primarily via an indirect paracrine effect rather than direct cell replacement in damaged areas [23]. As a result, researchers have shifted their focus toward the secretome of MSCs as an alternative cell-free (acellular) regenerative therapy to effectively promote wound healing through the stimulation of angiogenesis, modulation of inflammation, attenuation of scar formation, and induction of re-epithelialization [24,25].

The employment of the secretome (conditioned medium, CM) in in vitro and in vivo studies has revealed its potential for wound-healing therapy in both physiological and diabetic conditions [26]. Generally, it can be collected in vitro after cell exposure to serum-free medium or hypoxic conditions. The MSC-derived secretome comprises various soluble proteins such as growth factors (GFs), cytokines, and chemokines and molecules associated with extracellular vesicles (EVs) such as microvesicles (MVs; 100–1000 nm diam.), exosomes (40–150 nm diam.), and apoptotic bodies (1–5 µm diam.) [27,28,29,30].

**Figure 1:**
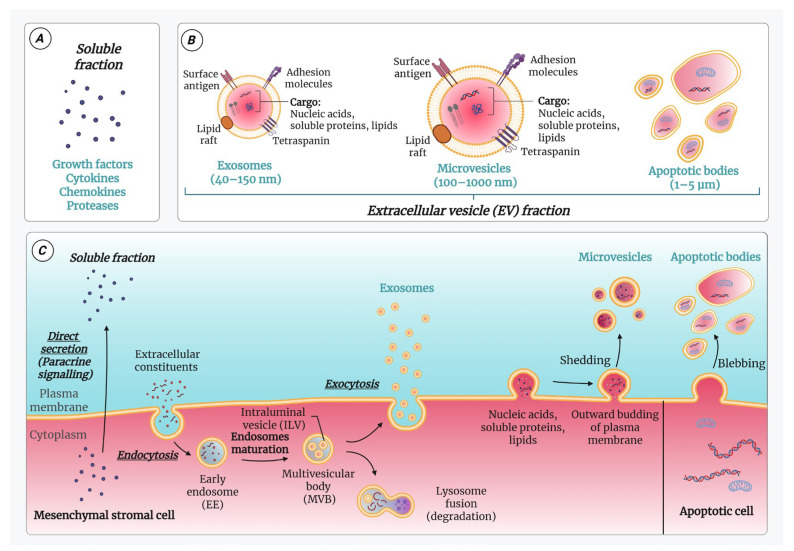
Illustration of the main secretome constituents: (**A**) the soluble fraction and (**B**) the extracellular vesicle (EV) fraction; the latter is made up of exosomes, microvesicles (MVs), and apoptotic bodies. The soluble fraction includes growth factors (GFs), cytokines, chemokines, and proteases. Within the surrounding bilayer membrane, EVs contain bioactive entities such as proteins, nucleic acids, and lipids. (**C**) The biogenesis pathways of the secretome components. Certain secretome constituents actively contribute to the wound-healing process by modulating inflammation, promoting cell proliferation, and supporting tissue remodelling. Image created with BioRender.com (accessed on 1 April 2025).

The soluble mediators are directly secreted through paracrine signalling pathways, meaning they diffuse into the immediate extracellular environment [31]. In terms of exosomes, considerable progress in unravelling the mechanisms underlying their formation and secretion has been made in the recent years. Briefly, the biogenesis of exosomes can be divided into different phases. First, various extracellular constituents along with cell surface proteins enter cells via endocytosis, resulting in the formation of the early endosome (EE) by the invagination of the plasma membrane. The next stage consists of the maturation of endosomes, during which their content is processed, characterized by the sorting and entrapment of proteins, lipids, and cytosol. This leads to the formation of multivesicular bodies (MVBs), with various intraluminal vesicles (ILVs) generated through the inward budding of the endosomal limiting membrane and enriched with specific cargoes (proteins, lipids, and genetic material). MVBs are then transported to the plasma membrane and dock on the luminal side of the cells. The last phase involves the fusion between MVBs and the plasma membrane, leading to the release of the ILVs as exosomes to the outside of the cells via exocytosis. Other MVBs fuse with lysosomes, where their content undergoes degradation and is released into the cytosol [32,33]. MVs are formed by the direct budding of the plasma membrane and contain proteins, lipids, and genetic material [34]. Apoptotic bodies, the largest class of EVs, are released during apoptosis via plasma membrane blebbing and consequently contain higher amounts of disintegrated organelles, in addition to the cytoplasmic proteins and nucleic acids [35,36]. The secretome constituents, their sizes, and their biogenesis are summarized in Figure 1.

Compared to cells, the cell-derived secretome has the advantages of being less immunogenic, having minimal risk of tumour formation [37,38], exhibiting enhanced safety, and being easier to handle [39]. For instance, exosomes remain stable during long-term frozen storage or at room temperature following lyophilization. Moreover, their small size further enables sterilization by filtration [40]. Foo et al. conducted an extensive comparison between stromal cells and the secretome, evaluating aspects such as manufacturing, quality control, the cost of production, and treatment [9].

According to the US National Institutes of Health website, various clinical trials have shown that the secretome can be safely administered with few reported adverse effects, often by intravenous injection or local injection, and may offer benefits in various applications including neurodegenerative diseases (e.g., phase I/II trial NCT00395200), wound healing (e.g., phase I clinical trial NCT04134676, phase I//I clinical trial NCT04277598), fistulizing Crohn’s disease (phase I clinical trial NCT05130983), and even COVID-19 (e.g., phase II clinical trial NCT04288102) (accessed on 27 May 2025) [22]. These findings are promising for the development of secretome-based products for clinical applications. Some companies involved in research and development (R&D) innovation have even created novel and highly effective products based on the secretome. These products are available on the market and utilize the secretome to address a range of skin conditions, including skin care (e.g., anti-ageing)—Carmell Secretome [41]; wound healing—Exostem4Tech manufactured by Falon Labs [42]; hyperpigmentation—Adiposecr product manufactured by Secretosome [43]; and psoriasis—NeoGenesis-patented S^2^RM [44]. One product supplemented with the secretome (NeoGenesis-patented S^3^RM) can address alopecia issues [45].

#### 2.2.1. Impact of Cell Culture Conditions on Soluble Fraction of MSC-Derived Secretome

**Figure 2:**
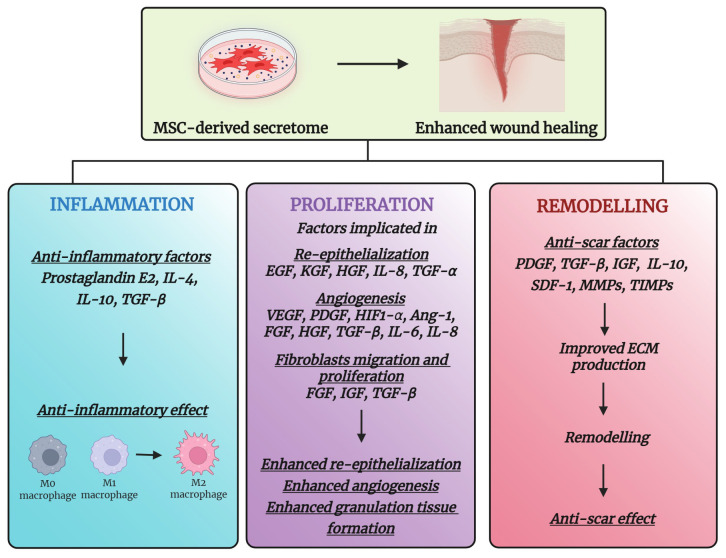
The regenerative capacity of the MSC-derived secretome in wound healing. Extracellular vesicles (EVs) and soluble factors (bioactive molecules) actively contribute to the different phases of wound healing, including inflammation, proliferation, and remodelling. Summarizing the main secreted molecules and their specific impacts throughout the healing phases [46,47], this figure highlights the comprehensive contributions of the MSC-derived secretome in promoting tissue repair. The abbreviations used in this figure are defined in the list at the end of this manuscript. Image created with BioRender.com (accessed on 19 November 2024).

The soluble fraction released by MSCs in the culture medium contains a wide variety of GFs and immunomodulatory molecules, cytokines, and chemokines that can affect different phases of the wound-healing process by acting on Fbs, keratinocytes, endothelial cells, and macrophages. Park et al. identified and quantified forty secreted proteins present in the ADSC-derived CM using a growth factor array. They identified elevated levels of epidermal growth factor (EGF), fibroblast growth factor-2 (FGF-2), and hepatocyte growth factor (HGF) [46], known to positively impact the proliferative phase of wound healing [47]. Reportedly, vascular endothelial growth factor (VEGF), HGF, platelet-derived growth factor (PDGF), transforming growth factor beta (TGF-β), FGF-2, angiopoietin-1 (Ang-1), Ang-2, and insulin-like growth factor (IGF-1) promote angiogenesis [48]. The relevant soluble factors and their associated effects on the phases of wound healing are summarized in Figure 2.

The efficiency of the MSC-derived CM depends heavily on the cell culture conditions. For example, varying the oxygen levels from normoxic to hypoxic conditions significantly affects the biomolecular profile of the CM. Park et al. reported that VEGF is significantly increased in ADSC-derived CM cultured in hypoxic conditions, while EGF levels are down-regulated (*p* < 0.05, Student’s *t*-test) [49].

Additionally, it was reported that MSCs cultured in three-dimensional (3D) systems secrete more beneficial biomolecules, including immunomodulatory factors. It is hypothesized that the 3D architecture provides a microenvironment closer to the tissue of origin, enhancing cell–cell communication and improving paracrine properties [50,51]. In this context, 3D culture can be achieved either through spheroid formation or by embedding cells in various biomaterials, primarily hydrogels [52]. Several studies have focused on harvesting the secretome from MSCs grown in 3D culture systems. Potapova et al. reported that MSCs grown in 3D spheroids secrete higher levels of paracrine biomolecules, such as VEGF and FGF-2, compared to MSCs cultured in monolayers [53]. Moreover, Kim et al. investigated the effects of ADSC-derived CM from a 3D culture (ADSC-CM-3D) on the migration and proliferation of keratinocytes and dermal fibroblasts (DFs). They reported that the secretome derived from the cells grown in the 3D culture was more effective in promoting the proliferation and migration of keratinocytes and DFs compared to CM derived from the two-dimensional culture (2D culture), suggesting distinct protein profiles in the two conditions [54]. More recently, Gangadaran et al. reported the angiogenic properties of CM harvested from bone marrow-derived MSCs (BM-MSC-CM-3D) grown as spheroids (3D culture). They evaluated the angiogenic properties in vitro by analysing the proliferation, migration, and tube formation capacity of endothelial cells as well as in vivo by assessing wound healing in a burn injury mouse model [55]. Wang et al. achieved the promotion of wound healing in rabbit third-degree burn models using a porous gelatine microcarrier crosslinked with hyaluronic acid and supplemented with polyethylene glycol thermogel as a dynamic 3D culture system for MSCs [56].

#### 2.2.2. Extracellular Vesicles: Nanocarriers Driving Wound-Healing Dynamics

EVs consist of a lipid bilayer enclosing a cargo that carries vital information and macromolecules from their source of origin, including lipids, proteins, DNA, and RNA molecules (such as mRNA and microRNA) [57]. These molecules contained within the EVs are capable of modulating recipient cells and are considered key mediators of the therapeutic potential associated with MSCs. Moreover, the protective lipid bilayer enables the effective delivery of the cargo, which contributes to the therapeutic effects [58]. Based on their morphological features and the mechanism of formation, the EVs exhibiting many of the therapeutic properties of the “parent” cells were classified into exosomes, MVs, and, to an extent, apoptotic bodies [28].

Exosomes, the smallest EVs, are membrane-bound vesicles released from almost all types of cells by an endosomal pathway and released into the extracellular space via the exocytosis process [59], as shown in Figure 1. Exosomes can be taken up and internalized by recipient cells through different mechanisms, subsequently exerting their complex biological effects [60]. For example, they play a significant role in a wide range of cellular functions, including intercellular communication, cell differentiation and proliferation, angiogenesis, the stress response, and immune signalling, through the delivery of diverse bioactive molecules [61].

Over the years, MSC-derived exosomes have been extensively analysed for their biological characteristics, functions, and potential clinical applications [9]. In the context of this review, studies have demonstrated that MSC-derived exosomes hold promise as a therapeutic approach for treating chronic wounds by stimulating the formation of new blood vessels, reducing inflammation, and promoting tissue remodelling [62]. It was shown that they contain cytokines such as VEGF, TGF-β1, interleukin 6 (IL-6), IL-10, and HGF, which are implicated in processes such as angiogenesis and inflammation following their internalization by recipient cells [63].

Although exosome-based therapies hold great promise for facilitating diabetic skin wound healing and regeneration, certain aspects require further optimization to enhance their effectiveness. These include identifying better cell sources, employing engineering techniques, adjusting the dosage and frequency, and integrating more efficient delivery methods. These strategies to maximize the therapeutic potential of exosomes are discussed in detail by Dong et al. in their recent review [64].

The remarkable potential of ADSC-derived exosomes in improving wound healing was demonstrated in in vitro and in vivo studies. However, this research is still in a preclinical stage, and its efficacy remains uncertain [65]. Yuan et al. conducted a systematic meta-analysis of preclinical animal studies and concluded that the administration of ADSC-derived exosomes significantly enhanced wound closure compared to controls (*p* < 0.001) [66]. Similarly, Wei et al. reported comparable findings focusing on diabetic conditions [67].

The therapeutic effect of ADSC-derived EVs could be further improved through various modifications (e.g., enriching exosomes with engineered non-coding RNA). Although promising, this approach requires further investigation [66]. Moreover, Song et al., using a diabetic mouse wound model, reported that incorporating ADSC-derived exosomes into an extracellular matrix (ECM)-based hydrogel accelerates wound healing by controlling exosome release [68]. In another study, Khalatbary et al. used ADSC-derived exosomes loaded into a bioengineered 3D amniotic micro-porous membrane scaffold, showing that this system accelerates diabetic wound healing [69].

Although exosomes and MVs share structural similarities, they differ in size, lipid composition, content, and origin. MVs are more heterogeneous than exosomes [70]. As illustrated in Figure 1, they are formed through the outward budding of the plasma membrane, a process that involves phospholipid reorganization and shedding [71,72,73].

Another type of EVs is apoptotic bodies, which are produced by apoptotic cells during the last stages of apoptosis [30,35]. Interestingly, apoptotic bodies are not just cellular debris and can influence the surrounding cells [74]. Nevertheless, their role in regenerative medicine remains largely unknown.

## 3. Fibroblast-Derived Secretome—Alternative Cell-Free Therapy for Chronic Wounds

### 3.1. Similarities Between MSCs and Fibroblasts

Fbs are stromal cells that provide for the majority of the structural framework—the extracellular matrix (ECM)—of almost all types of tissues, participating in tissue homeostasis. Their key role is the secretion of ECM components, including collagen fibres [14].

As reported, Fbs and MSCs share numerous common features, including a spindle-shaped morphology, localization within the connective tissue, multipotency or immune regulation, etc. (reviewed in [13,14]). Because of such similarities, Fbs could be used as a potential alternative to MSCs in regenerative medicine.

Additionally, Fbs possess some advantages that make them an attractive resource for wound-healing treatment, especially for secretome-based therapy. While the most commonly used MSC source, the BM, provides relatively little starting material for cellular expansion and requires invasive extraction methods, Fbs are easier to harvest in large numbers from various biological tissues such as skin, adipose, and gingival tissue. As suggested by Ichim et al., Fbs may provide an alternative to MSC use, based on their ease of isolation and cost-effectiveness. Moreover, the in vitro expansion of Fbs is significantly easier due to the robust nature of these cells, with a shorter population doubling time compared to MSCs [13].

Interestingly, the analysis of the transcriptome profile of DFs revealed that they maintain proliferative and secretory activity and genomic stability during in vitro culture in subsequent passages, at least up to the tenth one [75].

### 3.2. Constituents of Fibroblast-Derived Secretome

It has been reported that, like MSCs, cultured DFs secrete diverse wound-healing mediators into the culture medium. Notably, this production varies according to the composition of the medium. For example, the supplementation of FGF-2, EGF, and insulin in serum-free Fb medium resulted in a higher concentration of proteins relevant to the wound-healing process secreted by DFs in the culture medium [76].

The proteomic analysis of human DF-derived CM revealed that these cells secrete proteins involved in cell adhesion, attachment, proliferation, and migration, with potential wound-healing effects, at a higher concentration than those secreted by MSCs derived from Wharton Jelly [77]. More recently, numerous proteins in DF-derived CM that presented significant protein–protein interaction networks were identified. Proteomic and bioinformatic analyses revealed that these proteins are associated with wound repair and hair regeneration [78]. However, further studies are required to investigate their precise role.

In the CM derived from human nasal Fbs, proteins predicted to play important roles in wound healing were identified. Through mass spectrometry analysis, these proteins were divided into four classes, namely calcium-binding proteins, cell adhesion molecules, ECM proteins, and signalling molecules [79].

Interestingly, compared to constructs based on keratinocytes/Fbs only, DFs integrated into a fibrin-based construct and combined with keratinocytes (in a bilayer structure) showed better potential in stimulating the secretion of wound-healing mediators, which are important in the processes of inflammation, angiogenesis, re-epithelialization, and granulation tissue formation. More precisely, the bilayer substitute produced significantly higher amounts of various mediators, including CXC motif chemokine ligand 5 (CXCL5, *p* < 0.01), IL-6 (*p* < 0.05) and granulocyte colony-stimulating factor (G-CSF, *p* < 0.001), as determined by one-way analysis of variance (ANOVA). These molecules, once secreted following transplantation, may have a pivotal role in promoting wound healing [80]. Furthermore, Hu et al. showed that human DFs adhered to silk fibroin nonwovens released exosomes containing significantly higher amounts of angiogenic factors such as Ang-1, Ang-2, matrix metalloproteinase-1 (MMP-1), IL-1α, IL-4, and IL-8, compared to those derived from DFs cultured in a monolayer, as revealed through protein arrays. The GFs associated with exosomes isolated from scaffold-cultured DFs induced endothelial cells to form significantly more tubes in vitro (*p* < 0.05) [81]. Recently, it was demonstrated that the co-culture of human keratinocytes and Fbs on silk fibroin scaffolds enhanced the exosomal delivery of angiogenic GFs, including VEGF-A, FGF-2, IL-6, IL-8, MMP-9, tissue inhibitor of metalloproteinase-1 (TIMP-1), and TIMP-2 [82].

## 4. Experimental Models for the Assessment of Cutaneous Wound Healing

**Figure 3:**
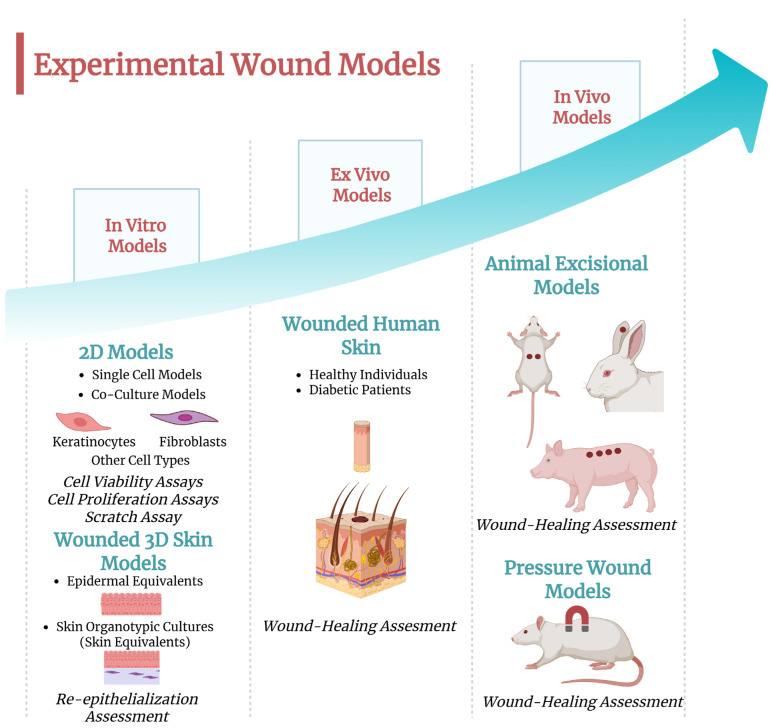
Schematic overview of experimental models used to study the wound-healing process, including in vitro, ex vivo, and in vivo models. The flowchart illustrates the progression from simplified in vitro systems to more complex in vivo environments. Image created with BioRender.com (accessed on 27 May 2025).

There is rich literature describing experimental models suitable for assessing skin wound healing, ranging from simple 2D in vitro to complex in vivo models [83]. Figure 3 illustrates the progressive relationship and increasing complexity of these models, ranging from in vitro to in vivo systems. Moreover, these models that serve as tools to evaluate the effects of different therapeutics are described below.

### 4.1. In Vitro Models of Cutaneous Wound Healing

**Figure 4:**
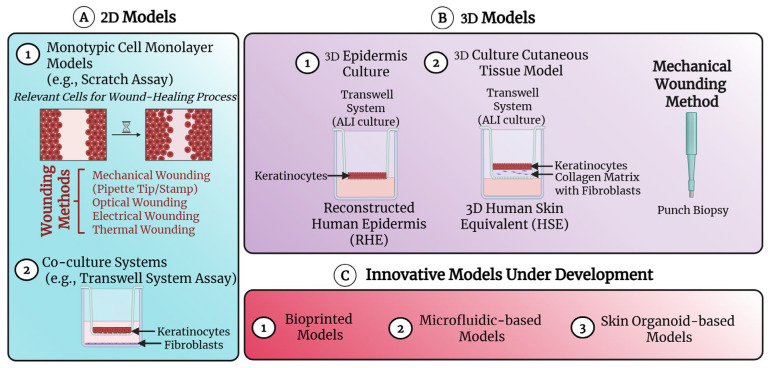
Schematic illustration of in vitro models used to investigate the process of wound healing and test novel therapeutic approaches. (**A**) Two-dimensional models: (**1**) The scratch assay consists of monitoring the wound-healing rate after wounding a cell monotypic monolayer (e.g., keratinocytes). (**2**) The Transwell co-culture system, where fibroblasts (Fbs) are cultured in the bottom well, and keratinocytes are grown on a porous insert. This setup allows the exchange of soluble factors between different cell types without direct contact. (**B**) Three-dimensional models: (**1**) The reconstructed human epidermis (RHE) model consists of stratified keratinocytes cultured at the air–liquid interface (ALI) on a collagen matrix placed on the porous membrane of a Transwell. (**2**) The human skin equivalent (HSE) model, known as an organotypic skin model, comprises keratinocytes cultured on top of a dermal equivalent composed of Fbs embedded in a matrix similar to the extracellular matrix. Various modalities are available to create wounds in 3D models, with the punch biopsy being the most commonly used method. (**C**) Innovative models under development: (**1**) Bioprinted models. (**2**) Microfluidic-based models. (**3**) Skin organoid-based models. Image created with BioRender.com (accessed on 21 July 2024).

In vitro models of wound healing can help address fundamental questions related to the cellular response to injury, allowing for the exploration of the molecular pathways involved [84]. These models are essential for testing novel therapy approaches, offering a cost-effective alternative and contributing to the reduction of in vivo experiments during preclinical evaluation. Currently, two major categories of in vitro wound models are available, 2D and 3D models, both of which are continuously being optimized (Figure 4).

#### 4.1.1. Two-Dimensional (2D) In Vitro Models of Cutaneous Wound Healing

Initially, cells involved in the wound-healing process were used in 2D culture systems to explore their roles in the different phases of skin repair, such as cell migration. The most commonly used method for analysing cell migration in a controlled environment is the scratch assay, a robust and replicable method [85,86]. Briefly, a cell-free area is created within a confluent cell layer (Figure 4). The mechanical method of wounding is most commonly employed, using tools such as a pipette tip. Other methods include optical, electrical, or thermal wounding [87]. The rate of cell migration into the scratched area is then determined microscopically [84]. These models are often developed using DFs, keratinocytes, and endothelial cells [23]. Moreover, Hodge et al. developed a diabetic wound model in vitro by inducing a diabetic-like phenotype in keratinocytes through high glucose supplementation in the medium. They used this model in a scratch assay to evaluate the potential benefits of the ADSC-derived secretome for treating diabetic wounds [88].

Beyond migration, other processes involved in wound healing can be studied using 2D monolayer cultures, such as cell proliferation, protein secretion, viability, gene expression, and differentiation. These experimental techniques allow for direct quantitative analyses, but they remain insufficient for fully evaluating new therapies.

In addition to monolayer cultures, another type of 2D model suitable for testing the efficacy of compounds in wound healing is the co-culture cell model based on a Transwell system. The latter facilitates communication between different cell types. Using this assay, the migratory potential of cells in response to chemotactic factors can be analysed [89]. A variety of cell types can be employed in this setup, providing more insightful data on cell–cell interactions compared to standard monolayer cultures. For example, this method allows the analysis of the keratinocyte–Fb crosstalk and interaction [90].

#### 4.1.2. Three-Dimensional (3D) In Vitro Models of Cutaneous Wound Healing

Compared to 2D cell cultures, 3D skin models enable more cell-to-cell and cell-to-ECM interactions. As a result, wound-healing tests using these models can better simulate wound physiology compared to 2D wound-healing assays [91].

Initially, the reconstructed human epidermis (RHE) model was developed by culturing keratinocytes on an inert matrix (Figure 4). These cultures are raised to the ALI (air–liquid interface), which triggers keratinocyte differentiation, leading to the formation of a stratified epithelium [92]. Later, some groups created models based on the co-culture of keratinocytes with Fbs using a two-chamber system. In these models, keratinocytes are cultured on a semi-permeable Transwell membrane at the ALI and allowed to differentiate, while Fbs are cultured in the well below. Soluble factors secreted by Fbs are able to pass through the membrane and affect the growth and differentiation of keratinocytes. These models were used to analyse the re-epithelialization rates and can also be employed to evaluate the effects of therapeutic agents [93].

To improve existing models, 3D in vitro skin organotypic culture models, also known as well as human skin equivalent (HSE), have been developed. These models allow for stratification, differentiation, and paracrine signalling between cell types (Figure 3). The models consist of a bilayer structure, combining dermal and epidermal components. The protocols for creating these models have been described [94,95]. Over the years, numerous attempts have been made to refine these in vitro skin models to more closely resemble the structure of the skin.

Many studies have investigated various methods for creating excisional wounds in these models to study re-epithelialization and obtain reliable results. Wounds can be created using biopsy punches, a scalpel, lasers, thermal wounding, or other techniques. Interestingly, Rossi et al. developed a device that generates automatically defined and precise wounds under sterile conditions [96]. This system minimizes the inadvertent detachment of the epidermal layer and ensures high reproducibility. However, wounding in 3D models is commonly carried out using mechanical techniques, such as biopsy punches. Safferling et al. standardized a 3D wounded-skin model using a biopsy punch, transferring the wounded construct onto an unpolymerized dermal compartment containing Fbs [97].

Basic in vitro models of the human skin, such as RHE and HSE, can now be produced in a standardized manner [98]. However, for wound-healing studies, the research community has focused on developing more relevant models. For example, incorporating macrophages and T cells could improve investigations of wound-healing phases and the evaluation of new therapeutic candidates [99]. In the recent years, several groups have developed immunocompetent skin models by integrating T cells [100,101]. However, it is important to recognize the limitations of these models due to the absence of the full spectrum of immune responses.

The in vitro modelling of wound-healing disorders is even more demanding, as ideal models need to reliably mimic the pathological phenotype, such as chronic wounds. Over the years, the research community has worked to develop models that replicate these wounds. For example, Maione et al. developed a diabetic model using diabetic foot ulcer-derived Fbs, which were encapsulated in a collagen type I matrix. The models showed several hallmarks of chronic ulcers, including impaired angiogenesis, re-epithelialization, and ECM deposition [102]. This study demonstrated that a skin model could accurately represent a pathological skin environment using patient-derived cellular components. More recently, Ozdogan et al. developed a thick pre-vascularized type 2 diabetic human skin model based on methacrylated gelatine. Cells were obtained from type 2 diabetic patients, and the model’s functionality was shown by applying a therapeutic hydrogel to the wound, promoting Fb migration [103]. However, the limitation of this model is the lack of epidermal differentiation and immune cells.

Interestingly, Kim et al. established a novel skin-mimicking model through a layered co-culture of stratified keratinocytes and Fbs, both differentiated from human induced pluripotent stem cells (iPSCs). This model, called skin organoid, could potentially be used for skin grafting and skin-related drug screening [104]. Moreover, it may be suitable for wound generation in vitro, though no reports have yet been published on this application.

Advancements in bioengineering techniques are enhancing the physiological relevance of 3D skin wound models. For example, 3D bioprinting has gained significant attention in recent years. Numerous 3D skin bioprinting models have been established, using different cell lines such as Fbs, keratinocytes, MSCs, or iPSCs [105,106]. Recently, Kang et al. reviewed the current advances and innovations in 3D-bioprinted skins, focusing on structural improvements such as hair follicles and vascularization as well as their wide application as drug screening platforms [107]. Compared to traditional skin models, 3D bioprinting offers many advantages including robustness, reproducibility, high resolution, and high-throughput culture [108]. One of the recent improvements in the bioprinting technique is associated with the use of silk fibroin, which is a natural biomacromolecule extracted from the cocoons of silkworms, with a high reported biocompatibility, adjustable biodegradability, and strong mechanical properties. In this context, Zhai et al. evaluated the effect of silk fibroin addition on the mechanical properties and rheology of the subsequent printed hydrogels, explored as an additive to enhance the pattern accuracy and fidelity of photo-triggered 3D printing [109]. This novel bioink recipe exhibits broad application prospects in the field of 3D bioprinting.

Three-dimensional-bioprinted skin constructs are considered suitable models for evaluating wound repair processes under various experimental conditions. For example, Douillet et al. recently developed an in vitro model that replicates fibroblast–myofibroblast dynamic remodelling [110]. Interestingly, Jara et al. developed a wounded 3D-bioprinted human skin equivalent with immune responses. They created wounds and printed a fibrin clot–macrophage bioink, achieving complete re-epithelialization. When compared with murine wound models, they concluded that this platform could replace in vivo models [111]. Sarmin et al. showed similar results using a decellularized porcine ECM-enhanced multicellular (DFs, immortalized keratinocytes, and monocytes) fibrin bioink [112]. Additionally, Kim et al. generated a diabetic bioprinted skin model that exhibited impaired wound healing. They observed typical pathophysiological hallmarks of type 2 diabetes, such as insulin resistance, adipocyte hypertrophy, inflammatory reactions, vascular dysfunction, and slow re-epithelialization after wounding [113].

In addition, following the global trend towards automating and integrating cell-based assays into microphysiological systems, conventional wound-healing in vitro assays have recently been improved using microfluidics and lab-on-a-chip technologies. The transition from static 3D cultures to dynamic 3D cultures has led to a better representation of human skin physiology, enabling direct drug studies [114]. Several studies have focused on developing skin-on-a chip models. For example, Ramadan and Ting developed an immune-competent model comprising keratinocytes and monocytes [115]. Additionally, skin-on-a-chip models based on macrophages co-cultured with Fbs, endothelial cells, and keratinocytes seeded on Matrigel were able to simulate the wound bed after tumour necrosis factor alpha (TNF-α) addition, with a reported increase in IL-6 and IL-8. These led to the disfunction of vascular structure organization, which was restored after treatment with dexamethasone [116]. These models also offer the advantage of using smaller amounts of reagents and cell materials. However, this field is still under development, and the hope is that these models can be used to test potential secretome-based therapies.

### 4.2. Ex Vivo Experimental Models of Cutaneous Wound Healing

Alternatively, several ex vivo human skin models have been developed to study skin tissue repair, especially re-epithelialization. Incisional or partial-thickness wounds are commonly used [117]. Gherardini et al. described an ex vivo model of wound healing that can be employed for testing specific promoting compounds [118]. Briefly, the model consists of creating a central wound using a biopsy punch that penetrates the epidermis and the upper part of the dermis. Similarly, Wilkinson et al. used a comparable model for diabetic skin and reported a reproducible method (based on the novel whole-mount staining technique) to accurately quantify the wound repair process [119].

Thus, the ex vivo wound model serves as an effective preclinical platform to evaluate the effects of the stromal cell-derived secretome. Despite the advantages of human ex vivo wound-healing models over in vitro systems, their widespread use is often constrained by the limited availability of skin biopsies and, in some countries, stringent ethical regulations.

### 4.3. In Vivo Experimental Models of Cutaneous Wound Healing

Compared to in vitro models, in vivo models offer a more realistic representation of the wound environment due to the presence of various cell types, surroundings signals, and paracrine interactions [120]. Often, they are used to evaluate the efficacy and safety of promising therapeutic agents before testing them in humans, an approach aligned with ethical regulations that restrict the participation of individuals with impaired wound healing in potentially harmful studies.

In vivo models typically involve creating wounds in experimental animals and monitoring wound closure over time using various methods (e.g., biochemical, histopathological, and immunological assays) [121]. Various wound models have been developed for both small and large animals, with mouse, rat, rabbit, and pig models being the most used [122]. Wounds can be experimentally induced through a variety of techniques; excision and incision, which allow the study of both acute and chronic wounds, are the most frequently employed. The in vivo wound models are described in the following subsections.

#### 4.3.1. Rodent Models of Cutaneous Wound Healing

Due to their physiological relevance to human wound healing and their feasibility in experimental settings, rodent models have emerged as valuable tools. In terms of wound repair, rodents have a much thinner epidermis and dermis compared to humans, so full-thickness models more closely mimic human wounds [123]. These models comprise the generation of wounds that completely remove both epidermal and dermal layers [124]. The most widely used species for generating these wound-healing models are rats and mice [121].

However, rodent models have been criticized for their translational relevance, having certain limitations. Firstly, in contrast to human wounds that heal via re-epithelialization and granulation tissue formation, the specific mechanism of wound closure in rodents is contraction. This process, which follows the injury, is due to the presence of a panniculus carnosus layer in rodent skin, consisting of a thin muscle layer [121]. To improve its relevance, the rodent wound model was enhanced with a splinted version. Specifically, Galiano et al. developed a splinted full-thickness model in mice, which effectively eliminates dermal contraction [125]. Another method to prevent wound healing through contraction is the rat tail excisional model where the splinting effect is exerted by the muscle and skeleton underneath the tail [126]. The wound is usually created as an excision on the dorsal part of the tail and requires 21 days for complete healing. Importantly, these wounds can be monitored and analysed without sacrificing the animals [127].

Another limitation of mouse models is the restricted number of wounds that can be created. In addition, due to the existing variability, these models require a reference wound for each mouse, increasing the number of animals required. Recently, Yampolsky et al. developed a reproducible strategy for excisional models in mice, providing a detailed procedure and guidelines, essential to decrease variability and obtain reproducible results [128].

To mimic chronic wound conditions, Hofmann et al. developed an ischemia-impaired wound-healing model in rats and demonstrated its capacity to test proangiogenic factors using a fibrin sealant matrix as a carrier [98].

Moreover, various diabetic murine wound models have been developed. The chemical ablation of pancreatic beta cells through streptozotocin injection (to model type 1 diabetes) was shown to delay the healing process in rats [129]. However, mouse diabetic models may not accurately reproduce the complexity of the diabetic process in humans. They resemble only one or few aspects of this complex disease [127] and typically only the early stages of diabetes [90]. Despite this, diabetic ulcer models in mice have shown a significant delay in wound healing compared with controls (wild-type mice), as recently reported based on a systematic review of 295 studies of mouse models combined with a network meta-analysis [130].

Models that mimic pressure wounds have also been developed in rodents, with rats being recognized as the most suitable. These models imply the utilization of pressure to induce insufficient blood supply to the skin, leading to hypoxia [131].

#### 4.3.2. Rabbit Ear Model of Cutaneous Wound Healing

The ears of rabbits are an attractive and cost-effective model for wound-healing studies. The ear skin architecture consists of the epidermis and dermis, which are attached to a cartilaginous layer that prevents contraction [127]. As a result, wound healing is promoted by re-epithelialization and granulation tissue formation [121].

Although the use of rabbit ear wound models is limited due to their restricted genetic traceability and the need for species-specific reagents [127], they are widely utilized. Recently, positive results were obtained in studies on the effects of silver nanoparticles in the wound-healing process [132]. Diabetic rabbit models are well established and can be chemically induced using alloxan [133]; they develop chronic wounds that are suitable for investigating impaired wound-healing processes [134].

The rabbit ear model is also widely used to study the impact of ischemia due to its complex vasculature that can be manipulated by ligating the vessels [135]. Like in humans, these large vessels tend to form collateral circulation, making the induced ischemia reversible [127].

Most animal models, including pigs and rodents, do not form hypertrophic scars, likely due to the fibromuscular layer found under the dermis [136]. It was reported that full-thickness hypertrophic scar wounds can be generated in the rabbit ear model [137].

#### 4.3.3. Porcine Models of Cutaneous Wound Healing

While the commonly used rodent and rabbit models provide valuable insights into skin wound healing, the translational relevance to humans often requires the use of larger animal models, despite their high cost, maintenance, and space requirements. Porcine models share significant similarities with human skin anatomy and physiology [138]. Therefore, full-thickness excisional wounds in pigs offer a closer representation of the human wound-healing process [139]. Moreover, pigs are a better choice for wound studies due to their larger dorsal surface, which allows for the generation of multiple wounds.

Currently, the pig wound model is well standardized and has been described as the most suitable preclinical model. Several gold standards were established for this model in accordance with the 3Rs principles [140].

Diabetic wound models in pigs have also been developed. Delayed re-epithelialization in diabetic pigs chemically induced with streptozotocin has been reported [141]. However, a limitation of the study is that the long-term effects of diabetes could not be assessed.

In addition, pigs have been used to simulate infected chronic wound models. Hirsch et al. developed an infected diabetic wound model in pigs [142], and recently, a porcine model with clinically relevant large infected chronic wounds was developed and used to assess a wearable electrostimulation bandage [143].

There are also challenges associated with pig models. Firstly, creating a large surface area of wounds could induce negative systemic effects or host responses that might impact the wound-healing process. As such, it would be difficult to characterize the systemic host response to different treatments, particularly those relevant to the immune system. As a result, rodent models may still be applicable for initial screening and testing due to their availability, cost, and convenience.

## 5. Wound-Healing Studies Using Stromal Cell-Derived Secretome

In this section, we will present the recent data obtained in vitro and in vivo using MSC- and Fb-derived secretomes.

### 5.1. Studies Demonstrating the Effect of MSC-Derived Secretome on Wound-Healing Process

#### 5.1.1. In Vitro Studies for the Evaluation of the Effects of MSC-Derived Secretome on Wound Healing

Various in vitro studies have demonstrated the effects of MSC-MC in supporting the wound-healing process. For example, it has been reported that ADSC-MC promotes the migration of skin cells—keratinocytes, Fbs, and endothelial cells [144]. Moreover, ADSC-derived CM effectively accelerates wound healing by stimulating the proliferative and migratory abilities of various skin cells, such as Fbs, keratinocytes, and endothelial cells, and wound contraction [23]. Importantly, the increased migration of keratinocytes in the presence of MSC-derived CM in a diabetes-like microenvironment was also detected [145].

Lombardi et al. summarized the data in this specific field, reviewing the effects of the ADSC-derived secretome on keratinocytes, Fbs, and endothelial and immune cells. While the comparative analysis of the results from different groups is challenging due to considerable variability, they concluded that the available findings support the use of cell-free therapies (i.e., the secretome) for the treatment of chronic wounds [146].

Recently, an ex vivo human skin wound model exhibiting the characteristics of low-perfusion chronic wounds was employed to compare the effects of ADSC-derived CM versus ADSCs. The authors reported that after topical application, both conditions were beneficial for re-epithelialization, which occurred at the wound edges, and improved the integrity of the ex vivo skin tissue [147].

#### 5.1.2. In Vivo Studies for the Evaluation of the Effects of MSC-Derived Secretome on Wound Healing

The beneficial effect of MSC-derived CM has been reported in experimental rodent models. Ma et al. compared the efficacy of locally injected ADSC-derived CM and ADSCs on wound healing using a full-thickness skin excision model in rats. In the early phase, a better therapeutic outcome consisting of the promotion of Fb proliferation and migration and the suppression of the inflammatory response was detected [148]. Likewise, Ahangar et al. showed that MSC-derived CM modulates inflammation and angiogenesis [149].

Interestingly, MSC-derived CM improved the wound healing of skin lesions in diabetic rats. Compared to the controls (non-treated wounds), the treatment of diabetic wounds with MSC-derived CM induces a significantly greater percentage of wound closure, less pronounced inflammatory responses, and increased angiogenesis [144].

An overwhelming number of studies based on rodent wound models have also demonstrated the efficacy of EVs in improving wound healing. Various rodent wound models used to assess the pro-wound-healing potential of ADSC-derived CM and ADSC-derived EVs have been reviewed by Lombardi and co-authors. Although the comparative analysis of the results from different groups is challenging (due to the variable experimental conditions), the available findings are encouraging [146]. However, more homogeneous in vivo studies are required to further demonstrate the clinical relevance of therapies based on the ADSC-derived secretome.

Recently, Prasai et al. performed a systematic review and a meta-analysis assessing the efficacy of exosome application in rodent wound-healing models. A total of 51 rodent studies were included, most of which reported the use of subcutaneous injection to deliver exosomes derived from stromal cells, primarily from BM, in both mice and rats. These exosomes were reported to regulate all phases of skin wound healing [150].

Regarding the in vivo assessment of the secretome’s role in wound-healing therapy, Hu et al. successfully utilized a full-thickness excisional ear model [151] following the classical protocol [152]. They used BM concentrate-induced MSC-derived CM and demonstrated its effects in increasing cell proliferation and complete wound closure on day 28 after its generation. Moreover, reportedly, the remodelling phase is modulated, ECM turnover is promoted, myofibroblast generation is inhibited, and profibrotic gene expression is decreased [151].

There is only a pilot study on wound healing based on the MSC-derived secretome in porcine models. The effect of ADSC-derived CM was assessed with a model of streptozotocin-induced diabetes. The CM and the cells were topically applied to the wound areas; the results revealed a statistically significant acceleration of wound closure compared with the control wounds (*p* < 0.05, Student’s *t*-test). Additionally, they observed an increase in angiogenesis and a reduction in the inflammatory profile after treatment with ADSC-derived CM, compared to the controls [153]. However, more studies are needed to determine the effects of the secretome on inflammation and angiogenesis during wound healing in diabetic swine.

#### 5.1.3. Clinical Studies for the Evaluation of the Effects of MSC-Derived Secretome on Wound Healing

Thus far, most studies have reported the therapeutic use of the MSC-derived secretome as an adjuvant therapy after cosmetic interventions; there are only a few reports indicating its effect on chronic wounds [154,155,156]. For example, in a randomized controlled trial, Prakoeswa et al. compared the efficacy of topically applied CM derived from human amniotic membrane-derived MSCs, with or without additional vitamin C and E, on chronic plantar ulcers in leprosy, over a period of up to eight weeks. They reported the safety and tolerability of the treatment along with a reduction in the mean ulcer size in all groups, with the greatest decrease observed in the group treated with CM and vitamin E [157]. In another randomized trial, Alinda et al. compared the efficacy of topically applied CM from ADSCs and human amniotic membrane-derived MSCs versus a farmazertin gauze dressing on the same type of wound, as novel therapeutic options. The results showed that ADSC-derived CM was superior to CM harvested from human amniotic membrane-derived MSCs and farmazertin only in terms of overall ulcer improvement [158].

Recently, MSC-derived CM was included in more clinical studies. According to the records from a global database of funded clinical studies, one completed phase I clinical trial has been registered, in which CM harvested from Wharton Jelly-derived MSCs was used for chronic ulcers (NCT04134676). The 38 patients enrolled were treated with the CM in gel form applied topically for two weeks. The primary results suggested a reduction in oedema and erythema, the formation of granulation tissue, and a decrease in the size of the ulcers [22].

Regarding exosomes, a pilot study ongoing in China (NCT05475418) involves the use of ADSC-derived exosomes combined with hydrogel as a dressing. Administration is conducted twice per week for four weeks, and the percentage of wound healing is monitored [22]. Unfortunately, to date, only five participants have been enrolled [22 January 2025].

### 5.2. Studies Demonstrating the Effects of Fibroblast-Derived Secretome on Wound-Healing Process

Considering its many molecular components, the secretome collected from Fbs provides a promising approach for chronic wound therapy development. Although various in vitro and in vivo studies have been conducted (see below), to date, no clinical trials using Fb-derived CM have been reported (22 January 2025).

#### 5.2.1. In Vitro Studies for the Evaluation of the Effects of Fb-Derived Secretome on Wound Healing

As early as 2012, Chowdhury et al. showed the effects of DF-derived CM on the expansion of keratinocytes by enhancing their attachment. Additionally, the analysis of keratinocytes’ growth kinetics showed that the supplementation with DF-derived CM obtained when keratinocyte medium was used is significantly more efficient than the condition using Fb medium [159]. Similar results in terms of keratinocyte attachment exposed to CM harvested from DFs cultured in keratinocyte medium were reported. However, according to the analysis of the keratinocyte wound-healing rate, the same CM induces a significant increase in keratinocyte migration due to higher calcium concentrations [160]. Moreover, it was shown that collagen mixed with DF-derived CM generated in Fb-specific medium exhibits a higher protein release compared to collagen with DF-derived CM isolated from DFs cultured in keratinocyte medium [161].

Chowdhury et al. explored the effect of DF-derived CM on skin wound healing and reported an improvement in the attachment and expansion capacity of keratinocytes [76]. Moreover, an enhancement in keratinocyte proliferation and migration was demonstrated in cells exposed to DF-derived CM in vitro [162].

Other sources of Fbs have been used to generate CM for the cell-free therapy of skin wound healing. For example, the effect of human gingival Fb-derived CM was compared to that of cell-based treatments. In vitro assessment revealed that gingival Fb-derived CM induces an improvement in the proliferation and migration of human Fbs, keratinocytes, and endothelial cells, as well as an enhancement in angiogenic function. They also identified growth factors and high levels of inflammation-related cytokines in the CM (through array analysis) that may contribute to these effects [163].

Interestingly, Topouzi et al. showed that CM collected from dermal papilla Fbs derived from hair follicles could accelerate re-epithelialization faster than interfollicular Fbs. They identified specific factors in the secretome derived from these cells responsible for the observed effect and demonstrated that these factors could also accelerate wound closure more than the currently used therapeutic PDGF in the clinic [164].

Different strategies to enhance the pro-healing actions of the secretome have been proposed. Hu et al. reported the enhanced angiogenic effects of exosomes containing GFs released by 3D-cultured DFs. They reported that the endothelial cells form a significant number of tubes in vitro and exhibit the enhanced secretion of various proteins [81].

In another study, a comparison between metabolically active and hypoxia-induced CM derived from BM-MSCs and DFs was performed. The authors found that the CM collected from both MSCs and DFs had high concentrations of angiogenic factors. However, the authors expressed a preference for DFs due to their easier accessibility, cost-effectiveness, and capacity to rapidly provide chemoattractive agents [165].

Similarly, Caneparo et al. used hypoxia (low-oxygen conditions) as a priming mechanism for cultured DFs before harvesting CM. They demonstrated that the hypoxia-exposed cells secreted soluble factors in the CM that significantly promote the proliferation and migration of endothelial cells and stimulate the formation and maturation of capillary-like structures, compared to a normoxic atmosphere or the specific EC commercial medium [166].

Related to the optimal collection timepoint for DF-derived CM, it was reported that the secretome collected after three days of incubation under serum-free conditions contained a higher protein concentration compared to that collected on earlier days. Moreover, the CM enhanced the in vitro re-epithelialization process by improving the keratinocytes’ attachment, proliferation, migration, and differentiation [160].

Recently, Fadilah et al. evaluated the potential effect of DF- and Wharton Jelly-derived MSC-derived CM collected across several cycles on Fb proliferation and migration and the protein release profiles. They showed that DF-derived CM collected over five repeated cycles significantly enhanced the Fb growth rate and migration compared to Wharton Jelly-derived MSC-derived CM. No differences were detected between the cell sources of CM regarding the protein release profiles [167]. Thus, DF-derived CM combined with a proper biomaterial constitutes a promising strategy for rapid wound-healing treatments. More than that, Maarof et al. developed an acellular skin substitute designed for the sustained release of essential mediators. This substitute consists of collagen hydrogels combined with chondroitin-4-sulfate and fortified with the DF-derived secretome [168]. The biocompatibility of the construct and its efficacy in therapy are yet to be assessed. The in vitro studies based on the role of the Fb-derived secretome in wound-healing therapy are summarized in Table 1.

#### 5.2.2. In Vivo Studies for the Evaluation of the Effects of Fb-Derived Secretome on Wound Healing

Maarof et al. developed an acellular 3D skin patch based on collagen hydrogel supplemented with DF-derived CM (DF-CM), and the construct was tested in vitro and in vivo. For the latter, a mouse full-thickness skin wound model was employed. In both tests, the construct did not induce an immune response. Furthermore, on day 7 after implantation, the DF-CM-supplemented collagen hydrogel induced a faster healing rate compared to the collagen-only group. These findings suggest that this construct could be employed as a potential off-the-shelf product for the immediate treatment of skin injuries [161].

Interestingly, gingival Fb-generated CM was evaluated for its pro-wound-healing effects using a murine excisional wound model. Both CM (acellular) and cell-based treatments significantly enhanced wound closure, a process associated with reduced inflammation, increased angiogenesis, and enhanced collagen deposition. Specifically, both conditions may confer beneficial gingival-like properties to dermal wounds [163].

Another in vivo study was performed on an ovine full-thickness wound model to evaluate an acellular skin patch made of collagen hydrogel with DF-CM. The results showed that the wound healed fastest in the presence of this construct, followed by a collagen sponge scaffold and a platelet-rich plasma gel, both combined with freshly harvested skin cells, indicating its suitability for the prompt treatment of full-thickness wounds [169]. The studies described above are summarized in Table 2.

## 6. Conclusions

MSCs have increasingly attracted significant scientific interest over the years, owing to their promising potential in regenerative medicine. However, there are several concerns about their possible side effects after direct cell transplantation, including immunocompatibility issues. It is well accepted that the beneficial effects of MSCs are mediated by the secretome rather than by cell replacement [23,170]. Hence, they can be utilized as an acellular therapy to treat various diseases, being a safe method [39].

The literature reviewed above highlights the considerable potential and importance of the cell-free (acellular) approach, namely the use of the secretome produced by MSCs and Fbs, for regenerative healing in chronic wounds. We focused on the impact of the cell culture conditions on the enhancement in the bioactivity of the MSC/Fb-derived secretome, a research area that continues to evolve. As was reported, the secretome derived from Fbs (accessible and cost-effective alternative to MSCs) was identified to have potential clinical applications, but this should be investigated further in more in vivo experimental studies, as well as in clinical studies.

Although MSC- and Fb-derived secretomes possess significant potential for clinical translation, various challenges must be addressed regarding donor variability and the standardization of protocols used for their generation in order to ensure reproducibility. Thus, the optimization method for secretome production and characterization represents a gap that needs to be addressed in this field. For example, more research into advanced culture systems and the optimization of production in bioreactors are essential steps. In addition, delivery systems using polymers for secretome administration are also under consideration in order to increase the treatment efficacy, offering greater stability, allowing controlled release rates and preventing off-target deliveries [170].

We also provide an overview of the currently available in vitro, ex vivo, and in vivo models used to assess different aspects of wound healing. These models serve as essential tools for evaluating the efficacy of acellular therapies before their translation to clinics. While no single model is superior to others, in vivo models are more representative of the complexity of the wound environment compared to in vitro models. Rodents are the most employed in vivo models due to their ease of use, cost-effectiveness, and reproducibility.

However, a suitable experimental model that fully recapitulates the conditions of chronic wounds is unavailable thus far. This limitation poses a translational challenge and highlights the need to integrate animal models with in vitro wound models and clinical studies to evaluate therapeutic compounds, including the stromal cell-derived secretome.

This field is expected to benefit from continuous advancement in technology. For example, recent developments in 3D bioprinting, microfluidics, and iPSC differentiation are paving the way for the creation of more sophisticated and standardized in vitro models. These advancements will facilitate the testing of novel therapies and improve the management of chronic wounds.

To summarize, the Fb-derived secretome has the potential to revolutionize wound treatment, supported by ongoing evidence from preclinical investigations.

## Figures and Tables

**Table 1:** In vitro studies showing the effects of the Fb-derived secretome on wound healing. The abbreviations used in this table are defined in the list at the end of this manuscript.

Fibroblast Type	Generation of Conditioned Medium (CM)/Exosomes/Acellular Skin Substitute	Effects on Wound Healing	Growth Factor Content	Ref.
Dermal fibroblasts (DFs)	Defined keratinocyte serum-free and Fb-specific serum-free media	Enhanced expansion of keratinocytes	Protein analysis not performed.	[159]
DFs	CM harvested after culturing the cells in hypoxic versus metabolic conditions	Similar efficiency to BM-MSC-derived CM in BM-MSC migration 2D assay	High concentrations of angiogenic factors. A higher number of angiogenic factors secreted compared to BM-MSCs.	[165]
DFs	Collagen mixed with CM harvested from cells grown in Fb and keratinocyte media	Higher protein release when Fb medium was used for CM generation	Protein analysis not performed.	[161]
DFs	CM harvested after culturing Fbs in keratinocyte-specific medium, Fb-specific medium with/without growth factors (GFs)—FGF, EGF, and insulin	Improvement in keratinocyte migration and expansion	A higher concentration of proteins and a consistent effect on the growth and migration rate when the Fb medium supplemented with GFs was used. Protein analysis not performed.	[76]
DFs	CM harvested after 72 h of incubation in keratinocyte- and Fb-specific media	Enhancement in keratinocyte proliferation and migration for all the concentrations used when Fb medium was used	Up-regulated genes that encoded proteins such as ECM structural constituents and remodelling enzymes, various GFs, cytokines, and chemokines or cell adhesion molecules.	[162]
Gingival Fbs	CM harvested after 24 h of incubation in Fb-specific medium	Enhanced proliferation and migration of human Fbs, keratinocytes, and endothelial cells and angiogenic function	The presence of FGF-2, HGF, VEGF, Ang-1, Ang-2, MMP-2, MMP-9, and TIMP-1. High levels of inflammation-related cytokines including IL-6 and IL-8.	[163]
DFs	CM obtained after exposure to hypoxia conditions for 1, 2, or 3 weeks—harvested three times a week and pooled	Promotion of endothelial cell proliferation and migration; stimulation of highly structured capillary-like network formation and maturation in fibrin gels	Overexpression of EGF and FGF-2. Up-regulated EGF, leptin, GM-CSF (granulocyte–macrophage colony-stimulating factor), and CXCL5 after exposure in hypoxic conditions. Unchanged amount of VEGF-A after exposure to hypoxia. PDGF-BB, Ang-1, and Ang-2 down-regulation.	[166]
Dermal papilla Fbs derived from hair follicles	CM harvested after 48 h of culture in keratinocyte medium	Dermal papilla Fbs derived from hair follicles accelerated re-epithelialization faster than interfollicular Fb subtypes, as shown by the scratch wound closure in vitro and punch wound closure ex vivo	Increased levels of cytokine sAXL (the soluble form of cell surface receptor tyrosine kinase) and CCL19 (chemokine ligand 19) when CM derived from dermal papilla Fbs was used.	[164]
DFs	Exosomes released by DFs cultured in a 3D system represented by silk fibroin nonwovens	Increased endothelial cell angiogenesis: a significantly increased number of tubes in vitro	Higher content of Ang-1, Ang-2, MMP-1, IL-1α, IL-4, and IL-8 present in the exosomal fraction of DFs cultured in 3D cultures, compared to those cultured in monolayers.	[81]
DFs	DF-derived CM added to acellular skin substitute based on collagen hydrogels mixed with chondroitin-4-sulphate	Suitable system for loading and delivery by slowly releasing essential mediators for wound healing from CM composition; the constructs maintain the characteristics of native collagen (swelling and degradation properties); the chondroitin-4-sulphate enhances the mechanical strength of the hydrogel; the efficacy in enhancing skin regeneration and its therapeutic application were not investigated	Protein analysis not performed.	[168]
DFs	CM collection after three days of incubation in serum-free conditions: defined keratinocyte serum-free and Fb-specific serum-free media	Enhanced attachment when keratinocyte-specific medium was used; enhanced re-epithelialization when Fb-specific serum-free medium was used; enhancement in keratinocyte attachment, proliferation, migration, and differentiation	12 wound-healing mediators detected (GFs, cytokines, and chemokines).	[160]
DFs	Five repeated cycles of CM collection from DFs versus Wharton Jelly-derived MSCs	Lower Fb attachment with CM from both cell sources compared to the control; increased Fb cell migration, compared to Wharton Jelly-derived MSCs-CM; similar Fb proliferation and protein release profiles to collagen hydrogel fortified with CM obtained from DFs and Wharton Jelly-derived MSCs	DFs released more proteins into the culture medium than Wharton Jelly-derived MSCs.	[167]

**Table 2:** In vivo studies depicting the effects of the Fb-derived secretome on wound healing. The abbreviations used in this table are defined in the list at the end of this manuscript.

Fibroblast Type	Generation of Conditioned Medium (CM)	In Vivo Model	Wound-Healing-Related Effects	Growth Factor Content	Ref.
Dermal fibroblasts (DFs)	DFs-CM-fortified collagen hydrogel (skin patch); CM harvested from DFs grown in Fb and keratinocyte media	Mice full-thickness skin wound model	DF-CM-supplemented collagen hydrogel (400 µg/mL total protein concentration) induced a faster healing rate than the collagen-only group on day 7 after implantation. The integrity and maturity of the regenerated skin (keratinocytes expressing cytokeratin 10 and involucrin revealed by immunostaining).	Protein analysis not performed	[161]
Gingival Fbs	CM harvested after 24 h of culture in serum-free medium; intradermal injection delivery around the margins of the wound	Murine excisional wound in vivo model	Reduction in average wound area and width on days 3, 7, and 14 of healing, accelerated re-epithelialization and resolution of inflammation, enhanced angiogenesis, and collagen deposition.	The presence of FGF-2, HGF, VEGF, Ang-1, Ang-2, MMP-2, MMP-9, and TIMP-1	[163]
DFs	Acellular skin patch made of collagen hydrogel with DF-CM versus collagen sponge scaffold with freshly harvested skin cells and a platelet-rich plasma gel with freshly harvested skin cells	Full-thickness wound ovine model	A thinner epidermis in all analysed conditions, as shown by histological evaluation. The integrity and maturity of the regenerated skin and keratinocytes expressing cytokeratin 10 and involucrin, as revealed by immunohistochemistry analysis. Fastest healing in the presence of DF-CM compared to the collagen scaffold (with freshly harvested skin cells) and controls.	Protein analysis not performed	[169]

## Data Availability

Not applicable.

## Abbreviations

The following abbreviations are used in this manuscript:

2D	Two-dimensional
3D	Three-dimensional
ADSCs	Adipose-derived stromal cells
Ang-1	Angiopoietin-1
ALI	Air–liquid interface
CXCL5	CXC motif chemokine ligand 5
CM	Conditioned medium
DFs	Dermal fibroblasts
ECM	Extracellular matrix
EGF	Epidermal growth factor
EE	Early endosome
EVs	Extracellular vesicles
Fbs	Fibroblasts
FGF-2	Fibroblast growth factor-2
GFs	Growth factors
G-CSF	Granulocyte colony-stimulating factor
GM-CSF	Granulocyte–macrophage colony-stimulating factor
HGF	Hepatocyte growth factor
HIF1-α	Hypoxia-inducible factor 1-alpha
HSE	Human skin equivalent
IGF-1	Insulin-like growth factor-1
IL-6	Interleukin 6
ILVs	Intraluminal vesicles
iPSCs	Induced pluripotent stem cells
KGF	Keratinocyte growth factor
MMP-1	Matrix metalloproteinase-1
MSCs	Mesenchymal stromal cells
MVBs	Multivesicular bodies
MVs	Microvesicles
PDGF	Platelet-derived growth factor
RHE	Reconstructed human epidermis
TGF-β	Transforming growth factor beta
SDF-1	Stromal cell-derived factor-1
TIMP-1	Tissue inhibitor of metalloproteinase-1
TNF-α	Tumor necrosis factor alpha
VEGF	Vascular endothelial growth factor
